# Pro-inflammatory endothelial cell dysfunction is associated with intersectin-1s down-regulation

**DOI:** 10.1186/1465-9921-12-46

**Published:** 2011-04-12

**Authors:** Sunit Singla, Dan Predescu, Cristina Bardita, Minhua Wang, Jian Zhang, Robert A Balk, Sanda Predescu

**Affiliations:** 1 Pulmonary and Critical Care Medicine, Rush University Medical Center, 1750 W. Harrison Street, 297 Jelke, Chicago, IL 60612, USA; 2 Department of Pharmacology, Rush University, 1735 W. Harrison Street, 1537 Jelke, Chicago, IL 60612, USA

## Abstract

**Background:**

The response of lung microvascular endothelial cells (ECs) to lipopolysaccharide (LPS) is central to the pathogenesis of lung injury. It is dual in nature, with one facet that is pro-inflammatory and another that is cyto-protective. In previous work, overexpression of the anti-apoptotic Bcl-X_L_ rescued ECs from apoptosis triggered by siRNA knockdown of intersectin-1s (ITSN-1s), a pro-survival protein crucial for ECs function. Here we further characterized the cyto-protective EC response to LPS and pro-inflammatory dysfunction.

**Methods and Results:**

Electron microscopy (EM) analyses of LPS-exposed ECs revealed an activated/dysfunctional phenotype, while a biotin assay for caveolae internalization followed by biochemical quantification indicated that LPS causes a 40% inhibition in biotin uptake compared to controls. Quantitative PCR and Western blotting were used to evaluate the mRNA and protein expression, respectively, for several regulatory proteins of intrinsic apoptosis, including ITSN-1s. The decrease in ITSN-1s mRNA and protein expression were countered by Bcl-X_L_ and survivin upregulation, as well as Bim downregulation, events thought to protect ECs from impending apoptosis. Absence of apoptosis was confirmed by TUNEL and lack of cytochrome c (cyt c) efflux from mitochondria. Moreover, LPS exposure caused induction and activation of inducible nitric oxide synthase (iNOS) and a mitochondrial variant (mtNOS), as well as augmented mitochondrial NO production as measured by an oxidation oxyhemoglobin (oxyHb) assay applied on mitochondrial-enriched fractions prepared from LPS-exposed ECs. Interestingly, expression of myc-ITSN-1s rescued caveolae endocytosis and reversed induction of iNOS expression.

**Conclusion:**

Our results suggest that ITSN-1s deficiency is relevant for the pro-inflammatory ECs dysfunction induced by LPS.

## Background

Severe sepsis is the leading clinical cause of lung injury [1]. No specific therapy currently exists for this critical illness, and efforts to reduce its burden have been limited by an incomplete understanding of the mechanisms of disease. Exposure of ECs to LPS is a widely used model for studying endothelial dysfunction in this syndrome [2]. The endothelial response to LPS is dual in nature, with one facet that is pro-inflammatory and another that is cyto-protective [2,3]. NF-κB and JNK-mediated pathways engage ECs to actively participate and regulate the pro-inflammatory process in response to LPS by expression and release of damaging components such as iNOS, cytokines, adhesion molecules, and pro-coagulants [4-6]. This response has been alternatively referred to as the activated endothelial phenotype [4,5]. Much less is known however, with respect to the exact nature, regulation and timing of the cyto-protective aspect of the ECs response. A role of Bcl members Bcl-2 and Bcl-X_L _in potentially shielding the cells from their proapoptotic environment has been described [2,3]. In our previous work, overexpression of Bcl-X_L _rescued lung ECs from apoptosis after siRNA-mediated knockdown of ITSN-1s, a novel scaffold and regulator of the general endocytic machinery, as well as a critical protein for pro-survival signaling in ECs [7,8]. ITSN-1s deficiency caused severe EC dysfunction, decrease in caveolae number, structural alterations and dysfunction of mitochondria, generation of mitochondrial reactive oxygen species (ROS) and apoptosis [7]. Similar observations of many of these phenomena in previous studies of LPS-induced endothelial dysfunction and the suggested role of Bcl-X_L _in cyto-protection during inflammatory stress led us to consider ITSN-1s as a participant in either of these events.

Numerous lines of evidence have documented the considerable oxidative and nitrosative stress associated with lung injury in human patients, and potential pathways by which either may mediate endothelial barrier dysfunction have also been well described [9,10]. iNOS is known to be up-regulated in response to LPS in a variety of cells, including lung ECs [11]. The resultant increased NO production has the ability to potentially alter a large variety of cellular processes such as interendothelial junctional integrity and thereby, basal vascular permeability [9]. The selective inhibition of iNOS has significantly attenuated the ability of endotoxin to generate lung injury in several animal models [12,13]. Notably, loss of caveolin-1 (cav-1) expression and thus lack of caveolae endocytosis and transcytosis resulted in constitutive activation of eNOS, increased NO production and a microvascular hyperpermeability cav-1 -/- mouse phenotype [14]. Thus, in the current study, we sought to further characterize ECs dysfunction and the elements of the cyto-protective ECs response to LPS.

## Methods

### Cell lines and reagents

Human lung microvascular ECs were purchased from Lonza (Walkersville, Inc., MD). LPS from Escherichia coli 011:B4, calmodulin from bovine testes, tetrahydrobiopterin (BH_4_), protease inhibitors cocktail, L-arginine and primers for qPCR were from Sigma-Aldrich (St. Louis, MO). Superoxide dismutase (SOD) from bovine erythrocytes was purchased from MP Biomedicals. Freeze dried Hb was from ICN Biomedicals. The *In Situ Cell Death Detection Kit, Fluorescein *was from Roche (Indianapolis, IN). Prolong Antifade Kit and neutrAvidin Alexa Fluor 594 were from Molecular Probes (Eugene, OR). EZ-Link Sulfo NHS-SS-Biotin was from Fisher Scientific (Hanover Park, IL). Streptavidin-HRP conjugated, MicroBCA (bicinchoninic acid) Protein Assay Reagent and Enhanced Chemiluminescent (ECL) Western Blotting Substrate were from Pierce (Rockford, IL). Specific antibodies (Abs) were obtained from the following sources: anti-Bcl-X_L _mAb, anti-NOS2 pAb from Santa Cruz Biotechnology (Santa Cruz, CA); anti-cyt c mAb from Calbiochem; anti-ITSN-1s mAb from BD Biosciences (San Jose, CA); anti-Bim pAbs from Millipore (Billerica, MA); anti-survivin pAbs from Novus Biologicals; Horseradish peroxidase (HRP)-conjugated reporters were from Cappel, Organon Teknika (Durham, NC).

### Endothelial Cell Culture and LPS treatment

ECs were cultured at 37°C in 5% CO2 in endothelial growth medium-2 obtained from Lonza and prepared according to manufacturer's instructions. Cells between the third and fifth passages were used for experiments. LPS (dry powder) was reconstituted in growth medium and applied over cells in a concentration of 1 μg/ml for a duration of up to 48 hours.

### Protein Extraction and Western blotting

Control ECs or cells exposed to 1 μg/ml LPS in culture media were collected from culture dishes and washed with PBS. Cell pellets were lysed for 1 h at 4°C in 50 mM Tris HCl, pH 8.0, 150 mM NaCl, 1% NP-40, and protease inhibitors. For detection of phospho-Bim (Ser^69^) levels, control and LPS-treated ECs were solubilized in kinase buffer (20 mM Tris/HCl, pH 7.4, 150 mM NaCl, 0.1% Nonidet P-40, 1% glycerol, 0.2 mM sodium vanadate, 0.83 mM benzamidine, 0.23 mM phenylmethylsulfonyl fluoride, 0.5 μg/ml aprotinin, and 0.5 μg/ml leupeptin). After centrifugation at 45000 rpm for 45 min at 4°C, the supernatants were collected and protein concentration determined by BCA with a bovine serum albumin standard. Equivalent protein amounts were subjected to SDS-PAGE and transferred to nitrocellulose membranes. The membranes were probed with anti-NOS2 pAb, anti-Bcl-X_L_ mAb, anti-ITSN-1 mAb, anti-Bim pAb, anti-survivin pAb, diluted in blocking buffer, (5% milk/TBS). Immunoreactive bands were visualized with the appropriate HRP-conjugated Abs and ECL detection. Densitometry was performed with ImageJ v1.37 software.

### Internalization Assay

Control and LPS-treated ECs, grown on plastic Petri dishes or coverslips were washed with ice-cold PBS and then incubated with 0.5 mg/ml cleavable biotin reagent as in [15,16]. Biotinylated cell surface proteins were internalized for 30 min, at 37°C. Biotinylated proteins still at the cell surface after 30 min were reduced with glutathione and the cells were further processed for morphological analysis or lysed for biochemical investigation. For biochemical studies, cells were lysed in TBS containing 2% Triton X-100, and the lysates were clarified by centrifugation for 30 min, at 4°C, 40,000 rpm in a TLA-45 Beckman rotor, to obtain a final supernatant expected to contain the internalized biotinylated proteins. The number of biotin molecules in the ECs lysates were quantified by ELISA as in [15]. Standard curves were generated using known concentrations of albumin-biotin. The average number of biotin molecules present in each cell lysate was determined at a series of decreasing concentrations from the linear part of the curve obtained by successively diluting a standard volume (100 μl) from each lysate and normalized per mg total protein. To obviate any interference between the biotinylated proteins internalized via caveolae and biotin present in mitochondria [17], we evaluated the biotin content of mitochondria by ELISA applied on lysate prepared from control ECs, not subjected to biotinylation of cell surface proteins. Mitochondrial proteins comprise only 0.25 × 10^14 ^biotin molecule/mg total protein, a value without statistical significance, when compared to the extent of biotin internalization via caveolae.

### Measurement of Transendothelial Electrical Resistance and paracellular permeability

ECs were grown to confluence on gold electrode array plates available from Applied Biophysics, Inc., Troy, NY. Transendothelial electrical resistance (TER) was measured by an electrical cell-substrate impedance sensing system (Applied Biophysics, Inc.) as described in [18]. Briefly, the gold electrode plates were connected to a phase-sensitive lock-in amplifier and a 1 volt 4000 Hz AC signal was applied through a 1 MΩ resistor. In-phase voltage was measured and used to determine TER which was normalized to initial values. A decrease in TER during the experiment reflects loss of cell-cell adhesion.

### Transwell assay

Transwell chambers with .4 μm pore filter inserts (BD Bioscience) were used. The inserts were coated overnight with 0.1% gelatin, at 37°C. Cells were seeded in the upper compartment and grown for additional 3 days post-confluency. Then, cell monolayers were subjected to 1 μg/ml LPS for 6 h, as an early time point, and 48 h, the time point used for biotin assay for caveolae internalization. The last hour of LPS treatment, 1 mg/ml dinitrophenylated (DNP)-BSA was added in the upper chamber. To quantify the transport, 500 μl medium from the lower chamber were removed and subjected to ELISA, via anti-DNP Ab, as previously described [19,20]. All measurements were performed in triplicate and repeated three times. A standard curve was generated by diluting known concentration of DNP-BSA.

### Molecular cloning of ITSN-1s expression construct

The full-length human ITSN-1s cDNA fragment (3,660 bp) was generated by PCR amplification from ITSN-1s cDNA (gift from Suzana la Luna, Center for Genomic Regulation, UPF, and Centro de Investigacion Biomedica en Red de Enfermedades Raras, Barcelona, Spain) with high-fidelity PCR enzyme (New England Biolabs), using the following primer pair: **ITSN1s_F269-BstBI**: 5' AGTA TTCGAA CC **ATG **GCT CAG TTT CCA ACA CCT T 3'and **ITSN1s_R3929-NheI**: 5' CGTA GCTAGC TTG CTG GCT TGG GTC CAT GTC TG 3'. The PCR products of the full-length ITSN-1s were digested with restriction enzyme BstBI and Nhe I (New England Biolabs) and purified (Qiagen purification kit), then cloned into a expression vector, pReiver-M10a (GeneCopoeia) at BstBI-NheI sites using T4 ligase (New England Biolabs). The ligation products were transformed into *E. coli *strain Top10 (Invitrogen). Dozens of transforming colonies were selected and subjected to PCR to identify the colonies with the ITSN-1s inserts using the same sequencing primer pair. The plasmid DNA was extracted from 6 selected growing clones bearing the ITSN-1s insert and was verified with correct restriction enzyme (BstBI and Nhe I) sites. Finally, one of them, CS-Z3999_ITSN1s #16 was selected for maxi-preparation of plasmid DNA, sequencing confirmation of the entire integrity of the full-length cDNA of ITSN-1s in frame in the vector and used for transfection of ECs. All transfections were performed using FuGENE 6, according to the manufacturer's instructions.

### Mitochondrial isolation and measurement of iNOS activity

#### Preparation of Mitochondrial fractions

EC monolayers grown on Petri dishes were washed three times with ice-cold PBS, pH 7.4, and collected by gentle scraping and centrifugation at 500 × g for 10 min. The cell pellets were resuspended in isolation buffer containing 250 mM sucrose, 40 mM Tris/HCl, pH 7.5, 10 mM MgCl_2_, 2 mM CaCl_2_, 1 mM phenylmethylsulfonyl fluoride, 15 μg/ml leupeptin as in [7]. They were kept on ice for 30 min and then disrupted with 40 strokes in a glass Dounce homogenizer. This was followed by centrifugation at 800 × g at 4°C for 10 minutes to remove unbroken cells, nuclei, and debris. The postnuclear supernatant was then subjected to centrifugation at 22,000 × g at 4°C for 10 minutes to isolate the mitochondrial pellet. The mitochondrial pellet was lysed in 50 mM HEPES, pH 7.4, 1% Nonidet P-40, 10% glycerol, 1 mM EDTA, 2 mM dithiothreitol, and protease inhibitors.

#### Spectrophotometric determination of iNOS activity

NO production was assayed by utilizing its reaction with oxyHb to produce the optically distinct methemoglobin (metHb) as previously described [21]. Briefly, unlysed mitochondrial pellets were resuspended in hypo-osmotic solution by adding 2 volumes of ice-chilled water containing a protease inhibitor cocktail. This was followed by sonication at 100 W, 50% duty cycle, and 75 s. Isolation buffer of twice the concentration used for the preparation of mitochondrial fractions was then added in the same amount as water used in the previous step. The suspension was centrifuged at 10,000 × g at 4°C for 10 min. The supernatant contained broken mitochondria and was used for the subsequent reaction.

Commercially available Hb contains a mixture of oxyHb and metHb which must be fully reduced to obtain pure oxyHb for the reaction. This was accomplished by the Dixon and McIntosh method [22]. Briefly, 0.2 ml of 0.1 g/ml sodium dithionite was added to a prepacked Sephadex column. When all of the dithionite had completely entered the gel bed, 1 ml of 50 mg/ml Hb was added. As the Hb passed through the dithionite zone, its color changed to purple and it then changed to an orange-red color as it left the dithionite. This was collected and the concentration measured spectrophotometrically using ε_415 nm _131.0 mM^-1^cm^-1^.

Dilution or oxidation of NOS substrates or cofactors during the preparation of broken mitochondria requires the presence of 5 μg/ml calmodulin, 50 μM L-arginine, 10 μM tetrahydrobiopterin (BH_4_), and 1 KU/ml SOD in the assay medium. These substrates were added to 1 mL of 100 mM HEPES, pH 7.1 in a cuvette followed by 4 μM oxyHb. 30 μg of mitochondrial protein was added and the optical density continuously recorded for five minutes at a wavelength of 401 nm. NO formation was quantified using ε_401(metHb-oxyHb) _of 49 mM^-1^cm^-1^.

### Terminal Deoxynucleotidyltransferase-mediated dUTP Nick End Labeling (TUNEL) Assay

Control ECs and cells exposed to LPS (1 μg/ml) for up to 48 h were washed in PBS and then fixed and permeabilized in methanol for 7 min, at -20°C. The TUNEL reaction mixtures (label and enzyme solutions) were prepared as directed by the manufacturer. It was applied over EC monolayers for 60 min, at 37°C, under dark and humid conditions. Monolayers exposed to label solution only were used as negative controls. The cells were washed and coverslips mounted using the Prolong Antifade kit. Fluorescence microscopy with an excitation wavelength of 488 nm identified TUNEL-positive cells.

### qPCR studies

Cells were lysed using the Qiagen QIAShredder homogenizer and the RNA was isolated using the Qiagen RNeasy Mini RNA isolation kit as per the manufacturer's instructions. cDNA was generated with the high-capacity cDNA RT kit by Applied Biosystems following the manufacturer's protocol. Amplifications were carried out using the following primers: ITSN-1s (5'-CAGGCTTGAAAGTCCTCAAAG-3', 5'-GGTGATCATGCTGGAAGTCA-3'), Bim (5'-TCCCTGCTGTCTCGATCCTC-3', 5'-GGTCTTCGGCTGCTTGGTAA-3'), Bcl-X_L _(5'-CTGTCGGTGGAAAGCGTAGA-3', 5'-TGCTGCATTGTTCCCATAGAG-3'), Survivin (5'-AAGAACTGGCCCTTCTT GGA-3', 5'-CAACCGGACGAATGCTTTT-3'). mRNA levels were quantified by real-time monitoring of the products of the cDNAs PCR with SYBR Green fluorescent dye. Cyclophilin was used as internal control.

### Electron microscopy

Control and LPS-treated cells were subjected to standard EM procedure [15]. Briefly, EC monolayers were fixed in 1.5% glutaraldehyde in 0.1 M sodium cacodylate buffer, pH 7.4, containing 5% sucrose for 30 min at RT. After washing in the same buffer the cells were post-fixed in Palade's 1% osmium tetroxide for 1 h on ice, stained with Kellenberger buffer, dehydrated in graded ethanol, and embedded in Epon at 60°C.

## Results and discussion

### LPS exposure causes an activated/dysfunctional ECs phenotype

**Figure 1:**
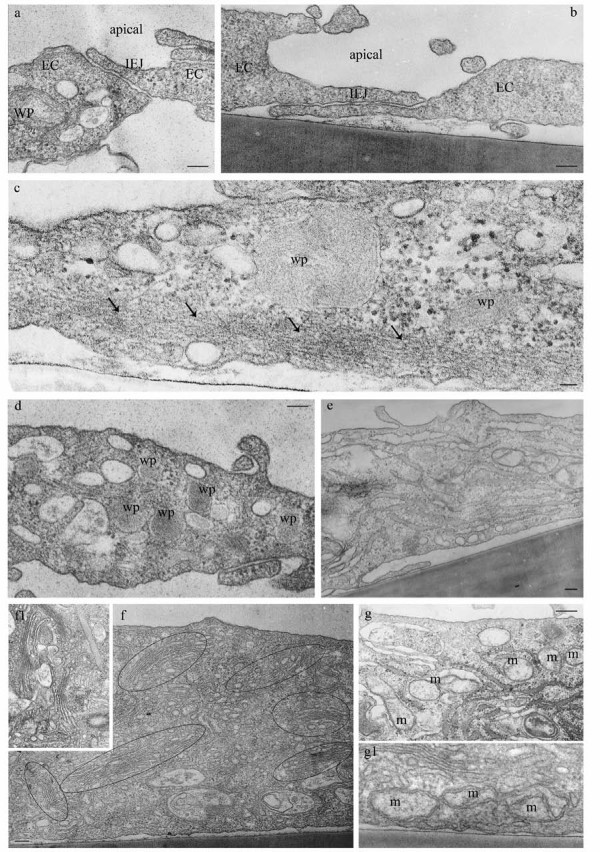
**LPS exposure causes an activated/dysfunctional ECs phenotype (I)**. ECs exposed to 1 μg/ml LPS for 48 h show open IEJs, (**a, b**), increased accumulation of actin filaments at cell periphery (**c, arrows**), significant increase in the number of Weibel-Palade (wp) bodies (**d**) and Golgi (**f, circled areas**). An enlarged and widespread endoplasmic reticulum network and the presence of a dilated tubular system are shown in **e**; frequently, increase number of lysosomal (lys) units is observed (**a**, **d, f**). Panel **g **shows a segment of cultured lung microvascular ECs comprising increased number of mitochondrial units (m), most of them with abnormal morphology. Note also the close association between ER and mitochondria. Frequently, mitochondria undergo a fission process (**g1**). Bars: 100 nm (a, b, d, e, f, g); 50 nm (c);

**Figure 2:**
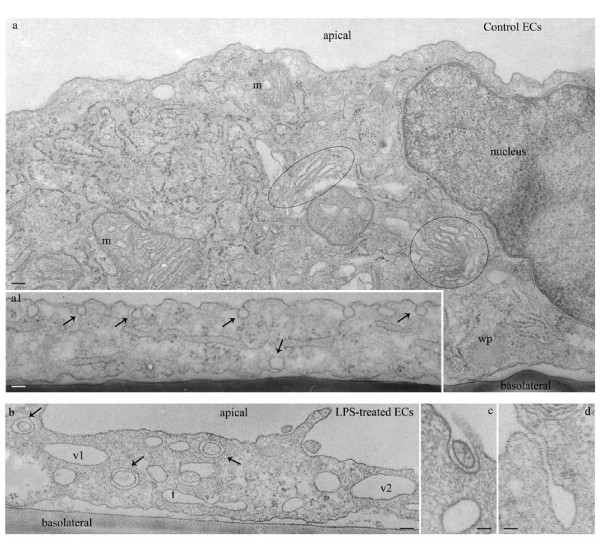
**LPS exposure causes an activated/dysfunctional ECs phenotype (II)**. Fragments of control ECs show a normal endothelial phenotype: Golgi stacks and vesicles as well as two mitochondrial units with well-organized cristae (**a**), a wide endoplasmic reticulum network is located within the two mitochondrial units. Note also a Weibel-Palade (wp) body in close proximity of the basolateral plasma membrane. Caveolae are open apically or basolaterally, apparently charging or discharging their load, (**a1, arrows**). In panel **b**, a fragment of an ECs subjected to 1 μg/ml LPS shows frequently large vacuoles (v1, v2), membranous rings, (**arrows**) and tubules (t), within cytosol or open to the cell surface (**c, d**), suggestive of deficient endocytic transport. Bars: 50 nm (a); 100 nm (a1); 70 nm (b); 40 nm (c, d).

It is well documented that ECs are active participants in and regulators of inflammatory processes such as severe bacterial infection [4,23]. However, there is still significant debate on the extent of vascular dysfunction that is due to the direct effects of bacterial products such as LPS on the endothelium. Thus, in this study we evaluated by high-resolution EM the morphological changes caused by direct LPS exposure on human lung microvascular ECs. Confluent ECs monolayers were treated with 1 μg LPS/ml, for 48 h, and then subjected to standard EM procedure as in [7]. Detailed examination revealed disrupted interendothelial junctions (IEJs), as illustrated in Figure 1a, b, and increased accumulation of actin filaments at cell periphery (Figure 1c arrows), suggesting increased paracellular permeability, one of the first sign of endothelial activation in response to inflammation. Several cellular organelles such as Weibel-Palade bodies and Golgi show apparently normal morphology, but their number was significantly increased (Figure 1d, f, f1) by reference to control ECs. Golgi organelles were spread throughout the cell, while a swollen endoplasmic reticulum (Figure 1c), occupied the entire intracellular space, observations consistent with expression of pro-inflammatory/cytoprotective genes as well as the ER stress. Fragments of control ECs are shown for comparison, (Figure 2a, a1). Note that these cells maintain in culture their endothelial phenotype; in close proximity of the nucleus there are Golgi stacks and vesicles as well as two mitochondrial units showing well-organized cristae. A normal, unswollen endoplasmic reticulum is detected within the two mitochondrial units. Note also a Weibel-Palade body by the basolateral plasma membrane. Caveolae, the main vesicular carriers of ECs, involved in the transport of plasma proteins and nutrients across the endothelium are open apically or basolaterally, apparently charging or discharging their load, (Figure 2a1 arrows). In LPS-treated ECs we have also frequently noted numerous small mitochondrial units. Large vacuoles, increased frequency of tubular structures and membraneous rings, (Figure 2b) some of them opened to the apical front of the cell (Figure 2c, d) are detected, suggesting impaired endocytosis and activation of alternative, non-conventional, transport pathways [24]. Altogether, these observations are consistent with an activated and dysfunctional EC phenotype.

### Endocytic dysfunction and a concomitant paracellular hyperpermeability is a hallmark of the LPS-EC phenoytpe

**Figure 3:**
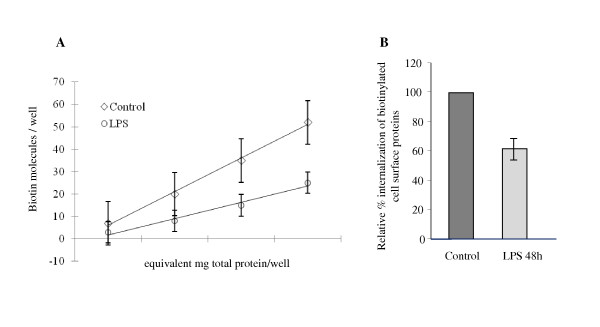
**Effects of 1 μg/ml LPS exposure causes significant inhibition of caveolae internalization**. **A**. Control and LPS-treated cells were subjected to biotinylation of cell surface proteins as described under Materials and Methods, followed by internalization of biotinylated proteins for 30 min, at 37°C. The number of biotin molecules present in lysates of control and LPS-treated cells was quantified by ELISA using streptavidin-HRP Ab, in 3 different experiments. The results were normalized per milligram total protein, per 30 min. Ordinate, amounts of biotin molecules/well detected per well; abscissa, μg total protein/well as result of serial dilution of control and LPS-treated ECs lysates. **B**. Degree of inhibition of caveolae-mediated uptake in LPS-treated ECs by reference to controls. Bars, ±SE.

**Figure 4:**
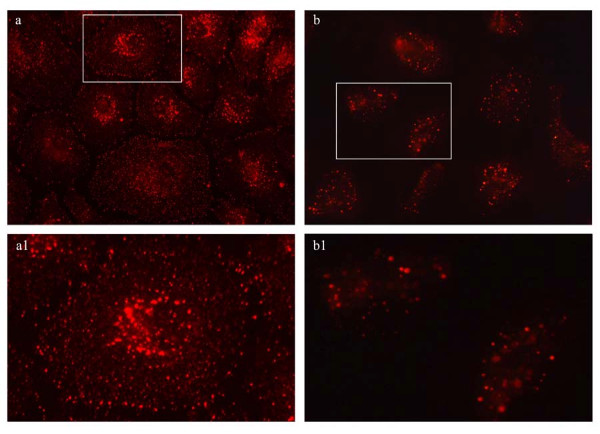
**NeutrAvidin Alexa Fluor 594 staining demonstrates inhibition of caveolae internalization by 1 μg/ml LPS exposure**. NeutrAvidin Alexa Fluor 594 staining of control ECs subjected to biotinylation of cell surface proteins followed by internalization assay, indicates a strong punctate pattern throughout the cytosol (**c**), with some accumulation in the perinuclear area. An enlarged ECs (boxed area in c) subjected to the internalization assay is shown in **c1**. ECs exposed to LPS and subjected to the internalization assay followed by neutrAvidin Alexa Fluor 594 staining (**b, b1**) show large fluorescent puncta within the intracellular space with no perinuclear accumulation. Bars: a, a1, b, b1 - 20 μm.

Since LPS signaling is regulated by caveolae endocytosis [25,26] and since EM analysis revealed endocytic abnormalities in LPS-treated cells, we next used a biotin internalization assay as in [15,16] to address if EC exposure to LPS affects caveolae endocytosis. This assay is a specific and reliable approach for addressing caveolae internalization in cultured ECs [15]. Caveolae-mediated uptake of biotinylated cell surface proteins is the dominant mechanism of internalization in ECs; the number of clathrin-coated vesicles in ECs is relatively small and their contribution to the internalization process is minor [27]. Briefly, control and LPS-exposed ECs were subjected to biotinylation of cell surface proteins, at 4°C, using a cleavable biotin reagent. Then, cells were transferred to 37°C, for 30 min, to allow internalization of biotinylated proteins. The biotinylated proteins, still on the cell surface after 30 min were reduced with glutathione. Then, the cells were either lysed for biochemical quantification of LPS effects on caveolae internalization by enzyme-linked immunosorbent assay (ELISA) or subjected to neutrAvidin-Alexa Fluor 594 staining for morphological surveys by fluorescence microscopy. Biochemical quantification of the number of biotin molecules internalized by control and LPS-exposed cells, Figure 3A indicated that in LPS-treated EC lysates, the number of biotin molecules was considerably smaller. A comparison of LPS-treated cells to controls shows an inhibition of 40%, (Figure 3B). Morphological analyses with NeutrAvidin-Alexa Fluor 594 revealed in control cells a fine punctate pattern throughout the cytoplasm, suggestive of vesicular association, (Figure 4c1). Frequently, biotin accumulation in the perinuclear area was detected. In LPS-exposed cells the staining was limited (Figure 4c2); large fluorescent puncta within the cytosol and no biotin accumulation in the perinuclear area were observed, suggestive of impaired biotin internalization. The intercellular spaces are large, consistent with disruption of IEJs. Highly magnified controlled and LPS-exposed cells (boxed areas), are shown, (Figure 4c1.1, c2.1).

**Figure 5:**
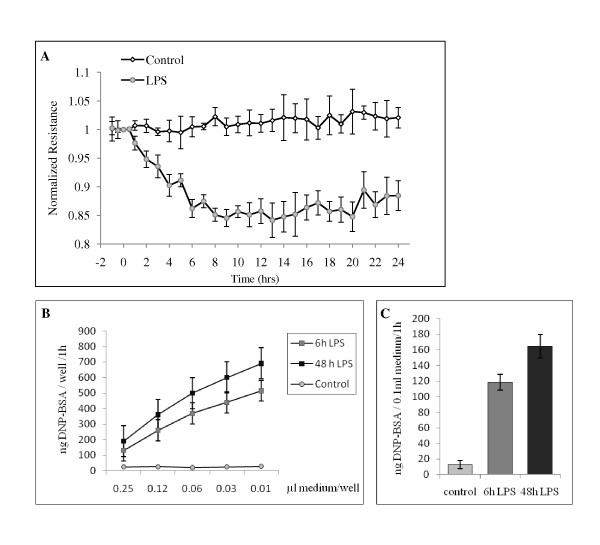
**1 μg/ml LPS exposure causes endothelial barrier dysfunction**. **A**. At the time point indicated (arrow), ECs grown on gold microelectrodes, were exposed to 1 μg/ml LPS, and TER was monitored over time. Data are expressed as means ± SD of 3 independent experiments; n = 3 per condition for each experiment. DNP-BSA concentrations detected in growth media collected from the lower chamber in control vs. LPS-treated ECs. Tracer amounts were determined by ELISA in 3 independent experiments, n = 3 per condition for each experiment. Ordinate, amounts of DNP-BSA detected per well; abscissa, μl growth medium per well. Degree of DNP-BSA leakage in LPS-treated ECs monolayer by reference to controls. Values are means ± SD ng DNP-BSA/0.1 ml growth medium/1 hour.

Since 1 μg/ml LPS exposure downregulates ITSN-1s expression and as a result transcellular transport is impaired, we next evaluated the status of paracellular permeability of ECs monolayers by measurements of TER, under the same conditions of LPS exposure. Monolayers of ECs grown on gold microlectrodes, three days post-confluency, were first stabilized for two hours to establish a linear TER baseline. Then, The stabilized monolayers were stimulated with LPS (Figure 5A, arrow) and TER was monitored over 24 hours, Figure 5A. A gradual decrease in the baseline barrier resistance was recorded for the first hours after LPS stimulation with a maximal decrease after 8 h and remaining in a plateau for the next 16 h hours. These data strongly suggest that LPS disrupts endothelial barrier and significantly increases the paracellular permeability of lung microvascular ECs. To get more insights on the extent of endothelial barrier dysfunction, we quantified the paracellular transport of dinitrophenylated albumin (DNP-BSA) using a transwell assay, as described under Methods. In brief, cells were seeded in the upper chamber and 3 days post-confluency, endothelial monolayers were subjected to 1 μg/ml LPS for 6 h, as an early time point, and 48 h, the time point used for biotin assay for caveolae internalization. In the last hour of LPS treatment, BSA-DNP was layered on top of EC monolayers to a final concentration of 1 mg/ml. To quantify the transport, 500 μl medium from the lower chamber were removed and subjected to ELISA, via DNP Ab, as previously described [19,20]. As shown in Figure 5B, the permeability of the endothelial monolayer for the DNP-BSA tracer were determined at a series of decreasing concentration from the linear part of the curve obtained by serial dilution of a standard volume of growth media removed from the lower chamber. In the presence of LPS, the amounts of DNP-BSA crossing the monolayer were considerably higher than control levels. Measurements of DNP-BSA amounts, using a series of concentrations on the straight part of the curve, indicated that at 6 hours after LPS exposure, the lower chamber contains 118.10 ± 7.3 ng DNP-BSA/100 μl medium, while at 48 h, 164.63 ± 12.4 ng DNP-BSA/100 μl medium, significant increase over the control levels estimated at 10.1 ± 0.9 ng DNP-BSA/100 μl medium, Figure 5C. The findings are consistent with endothelial barrier dysfunction, gap formation and DNP-BSA leakage, as result of LPS exposure.

### Induction and activation of iNOS and a mitochondrial variant by LPS exposure

**Figure 6:**
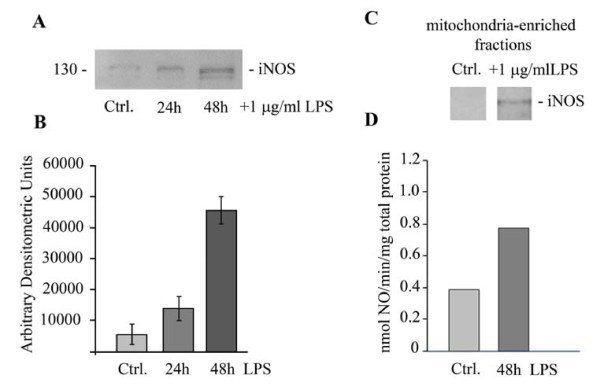
**Induction of iNOS and its mitochondrial variant expression by LPS exposure**. Total cell lysates (70 μg/lane) of control and LPS-treated ECs (24 h and 48 h) were subjected to SDS-PAGE, electrotransfer and immunoblotting with NOS2 Ab, known to recognize the iNOS and mtNOS [31]. A representative blot, documenting the increased expression of iNOS caused by LPS is shown in (**A**). Blots obtained from 3 different experiments performed under identical experimental conditions (70 μg total protein/lane, 1:1000 NOS2 pAb dilution and 30 sec ECL exposure time) were subjected to densitometric analysis (**B**), Bars, ±SE **C**. Control and LPS-treated ECs were subjected to cell fractionation to obtain an enriched-mitochondrial fraction; mitochondria were then lysed and analyzed by SDS-PAGE and Western blot using NOS2 Ab. **D**. mtNOS activity was assessed by exposing freshly prepared, unlysed mitochondrial fractions of control and LPS-treated ECs to oxyHb assay. The rate of NO production was measured by the change in optical density of the reaction solution as generated NO quickly oxidized oxyHb to metHb. The bar graph shows the difference in rate of mitochondrial NO formation between control and LPS-treated ECs.

Expression iNOS in response to LPS has been reported for several cell types such as epithelial cells, fibroblasts, hepatocytes, ECs [28,29]. A mitochondrial NOS, mtNOS, constitutively expressed, continuously active and associated with the inner mitochondrial membrane has been reported [21,30]. LPS-induced up-regulation of mitochondrial NO is one of the specific means, however still enigmatic, by which a dysfunctional and potentially pro-apoptotic environment is generated during the pro-inflammatory response in many cell types [31-33]. Thus, to evaluate the expression of iNOS and mtNOS in LPS-exposed human lung microvascular ECs, we first investigated by immunoblotting the expression of iNOS in control and LPS-treated ECs (Figure 6A). We detected weak iNOS immuno-reactivity under control conditions, and it increases by 27%, at 24 h and by 75% at 48 hours post LPS exposure (Figure 6B). Next, we investigated the effects of LPS on mtNOS expression. To this intent, mitochondria-enriched fractions prepared from these cells were lysed and subjected to SDS-PAGE. Even if mtNOS is constitutively expressed, the levels seem to be low, since no iNOS immunoreactivity was detected in the absence of LPS and under experimental conditions used (Figure 6C). However, in the mitochondrial fraction prepared from LPS-exposed ECs, mtNOS protein expression is easily detected, at 48 hours of LPS exposure (Figure 6C). Next, we addressed if the increased expression of mtNOS resulted in increased NO production. mtNOS activity was assessed by exposing freshly prepared, unlysed mitochondrial fractions from ECs to oxyHb, as described under Methods. The rate of NO production was measured by the change in optical density of the reaction mixture as generated NO quickly oxidized oxyHb to metHb. As shown in Figure 6D, mitochondria from LPS-treated ECs exhibited a two-fold greater rate of NO formation than untreated controls (0.81 nmol/min mg total protein versus 0.40 nmol/min mg total protein).

### ITSN-1s protein and mRNA levels are markedly decreased after LPS exposure

**Figure 7:**
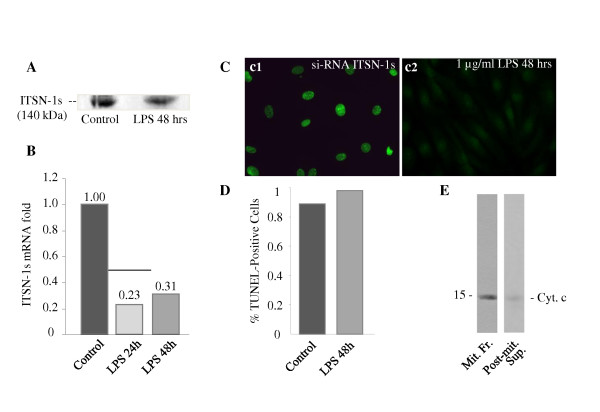
**LPS-induces ITSN-1s deficiency without affecting the EC survival**. Control and LPS-treated ECs lysates were analyzed by Western blot (**A**) and qPCR (**B**) for ITSN-1s protein and mRNA expression. The ITSN-1s mRNA levels, relative to the internal control, cyclophilin, were evaluated in three separate experiments. **C**. TUNEL staining of ECs treated with 1 μg/ml of LPS for 48 hours show no evidence for apoptotic activity (**c2**). A positive control - siRNA ITSN-1s - transfected ECs is shown in **c1**. **D**. Quantitative assessment was obtained by counting the number of TUNEL-positive and TUNEL-negative cells in 25 high-powered fields and calculating the percentage of apoptotic cells amongst the total cell population. No significant difference was noted in the percentage of apoptotic cells between LPS-treated and control ECs (0.89% vs. 0.98%, p = .45). **E**. Mitochondrial fractions and post-mitochondrial supernatants prepared for LPS-treated cells were analyzed for cyt c reactivity.

We have recently shown that specific and efficient ITSN-1s knockdown via a specific siRNA duplex targeting ITSN-1 gene results in severe EC dysfunction and apoptotic death, events that can be reversed by overexpression of the anti-apoptotic Bcl-X_L_[7]. Previous studies of LPS-induced endothelial dysfunction suggested the ability of LPS to induce pro-apoptotic pathways [34] as well as a role of Bcl-X_L _in cyto-protection during inflammatory stress [3]. These observations led us to consider ITSN-1s as a participant in either of these events. We therefore evaluated the levels of ITSN-1s protein in lysates prepared from LPS-treated and control ECs by immunoblotting with ITSN-1 pAb. The 140 kDa band representing ITSN-1s begins to decrease in intensity at 24 h and remains significantly decreased at 48 h after LPS exposure (Figure 7A). We also evaluated the level of ITSN-1s mRNA expression by quantitative PCR in ECs exposed to 1 μg/ml LPS for 24 or 48 hours. ITSN-1s mRNA levels were dramatically reduced in LPS-exposed ECs compared to control cells (Figure 7B). ITSN-1s mRNA expression was reduced 4.3-fold at 24 hours and 3.2-fold at 48 hours of LPS treatment compared to untreated controls. These results are consistent with the idea that LPS down-regulates the expression of the pro-survival protein ITSN-1s; ITSN-1s deficiency may be relevant to ECs dysfunction induced by LPS.

### Lung ECs do not undergo apoptosis in response to LPS

Since LPS-exposed ECs show decreased mRNA levels for ITSN-1s and since ITSN-1s deficiency is expected to initiate intrinsic apoptosis, we evaluated the occurrence of apoptosis in cells treated with LPS for 48 h, by TUNEL, Figure 7C, c1. Morphological surveys and morphometric analyzes show that the number of TUNEL-positive ECs is extremely low, comparable to untreated ECs (Figure 7D). A positive control, in which cells were subjected to ITSN-1s knockdown by siRNA as in our previous work [7], is shown in Figure 7C, c2. A complementary strategy to evaluate if mitochondrial cell death occurred was the examination of cyt c efflux from mitochondria. An enriched-mitochondrial fraction and the post-mitochondrial supernatant prepared from LPS-treated ECs normalized for total protein content were subjected to SDS-PAGE and Western blot with cyt c mAb. No immunoreactivity for cyt c was detected in the post-mitochondrial supernatant, consistent with cyt c confinement to mitochondria (Figure 7E). The findings suggest that ITSN-1s down-regulation as caused by LPS exposure of ECs did not trigger the intrinsic apoptotic pathway and suggest that LPS induces a cyto-protective response.

### LPS directly induces Bcl-X_L _and survivin expression, as well as down-regulation of the "BH3 only" Bim expression

**Figure 8:**
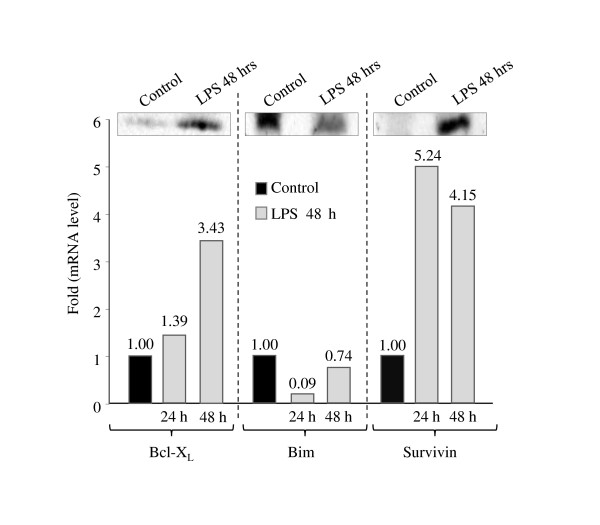
**Control and LPS-treated cell lysates (24 h, 48 h) were also analyzed by qPCR for mRNA expression of Bim, Bcl-X_L _and survivin, relative to the internal control, cyclophilin, in three separate experiments**. Representative results of one of these experiments are shown. Total protein concentration in control and LPS-treated cell lysates was determined by BCA, and 80 μg of total protein per sample was subjected to SDS-PAGE. Gels transferred to nitrocellulose membranes were then probed with Bcl-XL, Bim and survivin Abs. These trends were confirmed in at least three separate experiments for each protein.

The protective effects of Bcl-X_L _overexpression against LPS and ITSN-1s deficiency-induced cell death were previously documented [3,7]. However, whether LPS can directly induce Bcl-X_L _expression has not been reported for human lung microvascular ECs. To determine whether LPS up-regulates the mRNA for Bcl-X_L_, ECs were exposed to LPS for different time points (24 h, 48 h), and the levels of Bcl-X_L _protein and mRNA were determined (Figure 8). At 24 h, the levels of Bcl-X_L _are increased by 1.3-fold, while at 48 h a significant, 3.4 fold-increase, compared to controls is detected. Thus, LPS can up-regulate the mRNA for this cyto-protective protein. Levels of Bcl-X_L _protein correspond to mRNA accumulation. Interestingly, ECs deficient in ITSN-1s show decreased Erk1/2 activation [7]. Erk1/2 activation is responsible for constitutive phosphorylation of the pro-apoptotic "BH3 only" protein Bim, at Ser^69 ^leading to proteasomal degradation [35-37]. Thus, it is possible that down-regulation of Bim may be another possible mechanism by which ECs cyto-protection occurs in response to LPS. To determine whether LPS affects the levels of phospho-Bim (Ser^69^), control and LPS-treated ECs were solubilized in kinase buffer; equal amounts of total protein were subjected to SDS-PAGE and Western blot with a specific phospho-Bim (Ser^69^) Ab. Total Bim was detected with a specific anti-Bim pAb (Figure 8). Substantial decrease in both P-Bim (Ser^69^) and total Bim was noted. Next, qPCR for mRNA levels were performed; in response to LPS exposure, Bim mRNA was dramatically reduced, 11-fold compared to untreated controls at 24 hours and recovered to 1.2-fold reduction at 48 hours (Figure 8). These studies are consistent with the idea that down-regulation of Bim, as well as up-regulation of Bcl-X_L _serve as cyto-protective pathways against LPS-induced apoptosis in lung microvascular ECs.

Since Bim down-regulation was linked to the overexpression of survivin, an inhibitor of apoptosis protein, [38] and since LPS-induced NF-kB signaling may regulate survivin expression [39], we next examined by Western blot and qPCR the effects of LPS on survivin expression. As shown in Figure 8, survivin mRNA levels increased 5.2-fold in the first 24 h and remained increased by 4.2-fold at 48 h. The relative increase in survivin protein levels was confirmed by Western blot (Figure 8). The observation further documents that survivin up-regulation is another anti-apoptotic effect induced by LPS in ECs.

### Expression of myc-ITSN-1s in LPS-treated ECs restores endocytosis and inhibits LPS signaling

**Figure 9:**
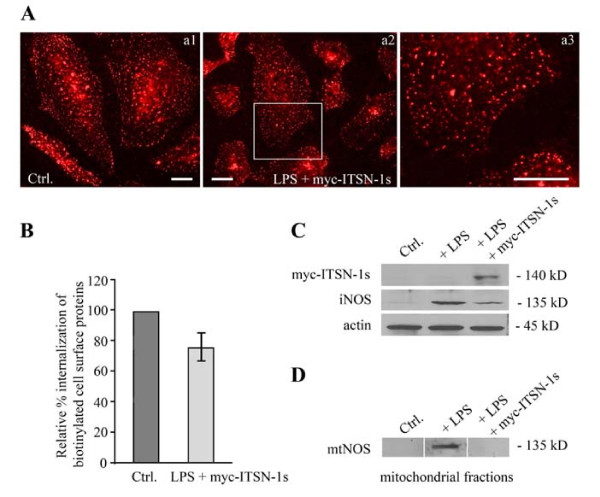
**Expression of myc-ITSN-1s in LPS-treated ECs restores endocytosis and inhibits both iNOS and mtNOS protein expression**. **A**. Myc-ITSN-1s-transfected ECs, 48 h post-transfection, were exposed to 1 μg/ml LPS cells for 48 h and then subjected to biotin internalization. NeutrAvidin Alexa Fluor 594 staining revealed the rescued ability of ECs to internalized biotin (**a2**). An enlarged region of interest (boxed area in a2) is shown in **a3**. Control ECs subjected to biotin internalization and NeutrAvidin Alexa Fluor 594 are shown for comparison in a1. Bars: a1, a2, a3 - 20 μm. **B**. Degree of inhibition of caveolae-mediated uptake in myc-ITSN-1s transfected and LPS-treated ECs, by comparison to controls was evaluated by ELISA in 3 different experiments. Bars, ±SD. **C**. Total ECs lysates (70 μg total protein/lane) prepared from control, LPS-treated and ITSN-1s transfected and LPS-treated cells were analyzed by SDS-PAGE, electrotransfer to nitrocellulose membranes and Western blotting for NOS2 protein expression. Actin was used as loading control. **D**. Enriched-mitochondrial fractions of control, LPS-treated and myc-ITSN-1s transfected/LPS-treated cells were analyzed as above for mtNOS expression.

Since cav-1 is a potential molecular chaperone that directly inactivates all NOS isoforms [40], and since ITSN-1s down-regulation inhibits caveolae internalization and thereby, increases LPS signaling [25], we sought to investigate if restoring endocytosis by ectopic expression of myc-ITSN-1s can affect iNOS expression. To this intent, subconfluent ECs monolayers, exposed to LPS for 48 h, were transfected with myc tagged full-length ITSN-1s, as previously described [15]. Efficient ITSN-1s expression was detected at 48 h post-transfection by immunoblotting of ECs lysates with anti-myc Ab, Figure 9C. Next, ECs grown on coverslips and exposed to LPS for 48 h, were transfected with myc-ITSN-1s and subjected to the biotin internalization assay. Fluorescent microscopy analyses of internalized biotin via neutrAvidin AlexaFluor 594 staining indicated that most of the cells display a fine punctate pattern staining (Figure 9A, a2, a3) similar to control cells (Figure 9A, a1), suggestive of biotin internalization via caveolae. Biochemical quantification of biotin molecules in lysate prepared from LPS-treated/ITSN-1s-transfected cells shows that these ECs significantly recovered their ability to internalize biotin, when compared to untreated cells (Figure 9B). Less than 25% inhibition of biotin internalization, still detected after myc-ITSN-1s expression, can be explained by the incomplete transfection of the entire ECs population. For the same reason, evaluation of the effects of ITSN-1s rescue on paracellular permeability is methodologically limited.

To evaluate the effects of myc-ITSN-1s expression and effective endocytosis on iNOS expression, we prepared total cell lysates from control, LPS-treated and LPS-treated/ITSN-1s-transfected ECs; equivalents of total protein amounts were analyzed by SDS-PAGE, electrotransfer to nitrocellulose membranes and western blot using NOS2 Ab. LPS-treated/ITSN-1s-transfected ECs show lower immunoreactivity to NOS-2 Ab and thereby, lower expression of iNOS, by comparison to LPS-treated ECs (no ITSN-1s transfection), Figure 9C. Then, the effects of myc-ITSN-1s expression on mtNOS in LPS-exposed ECs, were analyzed on mitochondrial-enriched fractions prepared from control, LPS-treated and LPS-treated/ITSN-1s-transfected ECs; mitochondrial lysates were normalized for total protein content and analyzed by SDS-PAGE, electrotransfer to nitrocellulose membranes and western blot using NOS2 Ab. As shown in Figure 9D, mtNOS is detected in the mitochondria fraction prepared from LPS-treated ECs. No NOS2 immunoreactivity was detected in control or LPS-treated/ITSN-1s-transfected ECs. The observation is consistent with the idea that ITSN-1s deficiency and the resultant impaired caveolae internalization is relevant to ECs response to inflammatory stimuli.

## Conclusions

Vascular barrier dysfunction contributes to the clinical hallmark of lung injury [41]. Transport of plasma proteins and solutes across the endothelium involves a cross-talk between the transcellular route via caveolae and the paracellular route through IEJs, [27,42]. In a previous study, when caveolae transcytosis was disrupted by cav-1 knockdown, the IEJs opened and a protein-rich interstitial edema reminiscent of LPS-induced lung injury developed [43]. Interestingly, treatment with the NOS inhibitor, L-NAME, reversed the effects of cav-1 knockdown on junctional hyperpermeability.

Our current observations of LPS-induced ITSN-1s down-regulation and subsequent endocytic traffic dysfunction and disruption of IEJs support the established cross-link between the transcellular and paracellular transport pathways and suggest a possible mechanism by which NO mediates this coupling is via NOS-2 activation and its association with mitochondria.

Our data also show that during LPS-induced ITSN-1s down-regulation, ECs do not undergo apoptosis as occurs when these cells are depleted of ITSN-1s via a specific siRNA duplex targeting ITSN-1s gene [7]. Furthermore, in our study we found that ECs may be protected from apoptosis by the compensatory LPS-mediated down-regulation of pro-apoptotic Bim and up-regulation of anti-apoptotic Bcl-X_L _and survivin. These observations are in line with previous studies indicating that human ECs undergo apoptosis in response to LPS only when protein synthesis is inhibited. Induced cyto-protection maintains ECs in a state of activated dysfunction which may perpetuate their critical contribution to the mechanisms of lung injury. Indeed, studies in animal models and human patients with lung injury have shown that apoptosis may be a prerequisite to the resolution of inflammation and initiation of repair [44,45]. One of the keys to advancing recovery in lung injury may therefore lie in understanding the transition between the dysfunctional, cyto-protective phenotype described here and the onset of programmed cell death.

Altogether, our studies established the role of the endocytic protein, ITSN-1s, as a novel node in LPS-EC signaling and offer interesting insights into the significance of endocytic dysfunction, mitochondrial NO production, cyto-protection, and the potential relationships between these systems in ECs during LPS-induced lung injury.

## List of Abbreviations

EC: human lung microvascular endothelial cell; ITSN-1s: intersectin 1-short; EM: electron microscopy; cyt c: cytochrome c; cav-1: caveolin-1; iNOS: inducible nitric oxide synthase; LPS: lipopolysaccharide; IEJ: interendothelial junction; mtNOS: mitochondrial nitric oxide synthase; DNP-BSA: dinitrophenylated bovine serum albumin; TER: transendothelial electrical resistance.

## Competing interests

The authors declare that they have no competing interests.

## Authors' contributions

SS and SP conceived of the study, designed the experiments, and drafted the manuscript. DP performed the electron microscopy, TER measurements, and assisted in experimental design throughout the study. SS and CB performed the Western blotting, and immunofluorescence studies. CB carried out the ELISA for the biotin internalization and DNP-BSA assays. MW generated the ITSN-1s construct and JZ designed the primers for and carried out the quantitative PCR experiments. RAB participated in coordinating the study and helped draft the manuscript. All authors read and approved the final manuscript.
